# Role of FOXO1 in aldosterone-induced autophagy: A compensatory protective mechanism related to podocyte injury

**DOI:** 10.18632/oncotarget.9644

**Published:** 2016-05-26

**Authors:** Bin Wang, Wei Ding, Minmin Zhang, Hongmei Li, Honglei Guo, Lilu Lin, Jing Chen, Yong Gu

**Affiliations:** ^1^ Division of Nephrology, Huashan Hospital and Institute of Nephrology, Fudan University, Shanghai, China; ^2^ Division of Nephrology, Shanghai Ninth People's Hospital, School of Medicine, Shanghai Jiaotong University, Shanghai, China; ^3^ Division of Nephrology, The Fifth People's Hospital of Shanghai, Fudan University, Shanghai, China

**Keywords:** podocyte, aldosterone, autophagy, FOXO1, apoptosis

## Abstract

This study was undertaken to elucidate whether and how autophagy was regulated in aldosterone (Aldo)-induced podocyte injury and to examine its role in this model both *in vitro* and *in vivo*. In cultured podocytes, Aldo increased autophagy flux as indicated by the enhanced expression of LC3-II/LC3-I and the reduction of p62. Autophagy induction with rapamycin (RP) provided a cytoprotective effect, and inhibition of autophagy with Atg7-specific siRNA, chloroquine (CQ) or 3-methyladenine (3-MA) worsened Aldo-induced podocyte injury by attenuating endoplasmic reticulum (ER) stress. Aldo inhibited Akt phosphorylation but increased the mammalian target of rapamycin (mTOR) signaling pathway; however, Aldo up-regulated the expression of FOXO1 and its downstream effector Rab7. Either knockdown of FOXO1 or Rab7 inhibited Aldo-induced autophagy. Additionally, an elevated level of P300-regulated acetylation of FOXO1 and the interaction of acetylated FOXO1 and Atg7 were also confirmed to be involved in regulating autophagy in Aldo-induced podocytes. Similar results were further confirmed *in vivo*. We propose that autophagy enhancement through enhancing of the FOXO1/Rab7 axis and post-translational modification of FOXO1 may represent a potential therapeutic strategy against podocyte injury by promoting autophagy.

## INTRODUCTION

Podocytes are terminally differentiated cells that line the urinary side of the glomerular basement membrane. Growing evidence suggests that podocyte injury leads to proteinuria and plays a crucial role in the progression of end stage renal disease [1, 2]. The activation of the renin-angiotensin-aldosterone (Aldo) system (RAAS) is a major hallmark in the development and progression of organ damage in chronic kidney diseases (CKD). Recently, accumulating evidence has suggested that Aldo, originally produced in the glomerulosa zone of the adrenal cortex, also plays an important role in the pathogenesis of podocyte injury [3, 4].

Endoplasmic reticulum (ER) stress is characterized by the increased expression of ER chaperones, the translational arrest of protein synthesis in the ER, and the stimulation of ER-associated degradation. Accumulating evidence indicates that ER stress can result from various disturbances, including hypoxia or oxidative stress and the activation of inflammatory signaling, processes that are also known to be involved in the pathogenesis of Aldo-induced kidney injury [5–7].

Macroautophagy (hereafter referred to as autophagy) is an evolutionarily conserved catabolic mechanism to maintain energy homeostasis and to remove damaged cellular components [8, 9]. Impaired autophagy can result in overt ER stress [10]. Autophagy has also been implicated in the physiologic and pathophysiologic processes of many kidney diseases, including diabetic nephropathy [11], IgA nephropathy [12], and aging kidneys [13]. Up-regulated autophagy can limit the effects of overt ER stress in response to cell injury [14, 15]. Previous studies have demonstrated that ER stress and autophagy were involved in Aldo-induced podocyte injury [16]. However, the relationship between these processes and how autophagy is regulated in Aldo-induced podocyte injury is not clear.

Mammalian target of rapamycin (mTOR) signaling is a master negative regulator of autophagy, which inhibits autophagy by inhibiting Atg1 from recruiting its partners Atg13 and Atg17. The mTOR inhibitor rapamycin (RP) induces autophagy in many cell systems [17]. FOXO1 (also known as forkhead in rhabdomyosarcoma, or FKHR) is a member of the forkhead box O (FOXO) family. Recent evidence has revealed an important role for FOXO1 in the regulation of autophagy given that FOXO1 promotes the expression of several autophagy-related genes and that post-translational modifications of FOXO1 participate in the autophagic process [18–20]. Both mTOR and FOXO1 are the main downstream effectors of the Akt pathway [21], which is inhibited by treatment with Aldo in podocytes [22].

In the current study, we demonstrated that ER stress is involved in Aldo-induced podocyte injury and that Aldo is able to induce autophagy in podocytes through the up-regulation of the FOXO1/Rab7 axis and the acetylation of FOXO1 rather than inhibiting the classic Akt-mTOR pathway. We propose that autophagy enhancement through the regulation of FOXO1 may represent a potential therapeutic strategy against podocyte injury both *in vivo* and *in vitro*.

## RESULTS

### ER stress is involved in Aldo-induced podocyte injury

**Figure 1:**
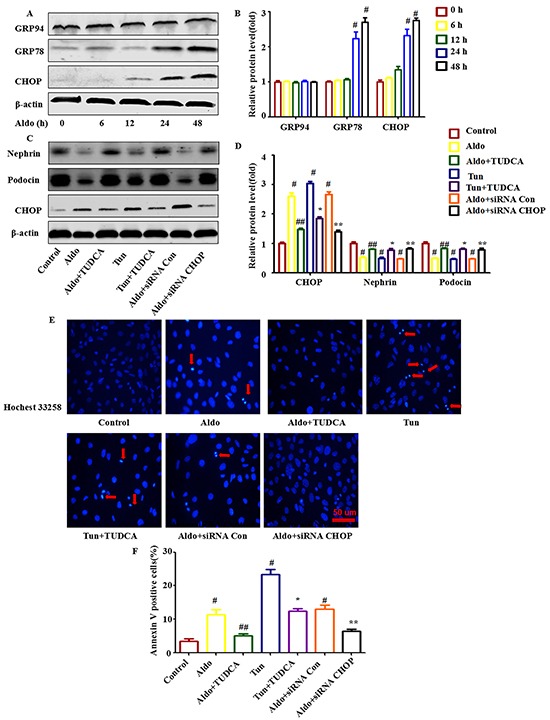
ERS is involved in Aldo-induced podocyte injury in cultured podocytes **A.** Western blot analysis showed the expression of CHOP, GRP78, GRP94, and β-actin proteins in podocytes after treatment without or with 10-7 M Aldo for various time periods, as indicated. The same blot was re-probed with β-actin to ensure equal loading of each lane. **B.** Graphical presentation shows the relative abundance levels of CHOP, GRP78, and GRP94 after normalization with β-actin. **C.** Equal numbers of podocytes were incubated in media containing buffer (control), taurine-conjugated derivative (TUDCA; 100 μM), tunicamycin (Tun; 5 μg/ml), siRNA-Con (20 nM) or siRNA-CHOP (20 nM) with or without Aldo (10-7 M) for 48 h, as indicated. The whole cell lysate was immunoblotted with antibodies against Nephrin, Podocin, CHOP, and β-actin. **D.** Graphical presentation shows the relative abundance levels of Nephrin, Podocin, and CHOP after normalization with β-actin. **E.** Hochest33258 staining after various treatments, as indicated. The red arrow indicates apoptosis-induced chromatin condensation and fragmentation. Scale bar=50 μm. **F.** After the various indicated treatments, podocytes were stained with Annexin V and propidium iodide (PI) and were then analyzed by flow cytometry. Quantification of apoptotic cells by flow cytometry. Results (means±SEM) of 3 series of experiments. #P<0.05 vs. normal control, ##P<0.05 vs. Aldo alone, *P<0.05 vs. Tun alone, **P<0.05 vs. Aldo+siRNA Control.

To analyze ER stress, we examined ER stress markers in Aldo-induced podocytes. As shown in Figure 1A and 1B, upon exposure to 10^−7^ M Aldo for various times (as indicated), the GRP78 and C/EBP homologous protein (CHOP) expression levels, which are typically up-regulated during severe ER stress, were up-regulated distinctly as early as 24 h following the initiation of Aldo stimulation and showed the greatest accumulation at 48 h following Aldo stimulation. However, Aldo had no significant effect on the expression of another ER stress marker, GRP94 (Figure 1A through 1B).

To further characterize the role of ER stress in Aldo-induced podocyte injury, we then treated cells with tunicamycin (Tun), an N-acetyl glycosylation inhibitor, as a positive control. Compared with the control cells, both Tun and Aldo increased ER stress and podocyte injury, as indicated by the increased expression level of the ER stress marker CHOP (Figure 1C and 1D), the number of apoptotic podocytes (Figure 1E and 1F), and the reduced expression levels of the podocyte makers Nephrin and Podocin (Figure 1C and 1D). However, pretreatment with the chemical chaperone TUDCA, an ER stress inhibitor, for 1 h attenuated Aldo- or Tun-induced ER stress and podocyte injury (Figure 1C through 1F). To further confirm the role of ER stress in Aldo-induced podocyte injury, podocytes were transfected with CHOP siRNA. In these podocytes, CHOP expression was significantly increased following Aldo or Tun treatment (Figure 1C and 1D), and CHOP inhibition significantly reduced podocyte injury (Figure 1C through 1F). These data suggest that Aldo-induced podocyte injury is mediated at least partially through ER stress.

### Aldo increases autophagic flux earlier than ER stress in cultured podocytes

**Figure 2:**
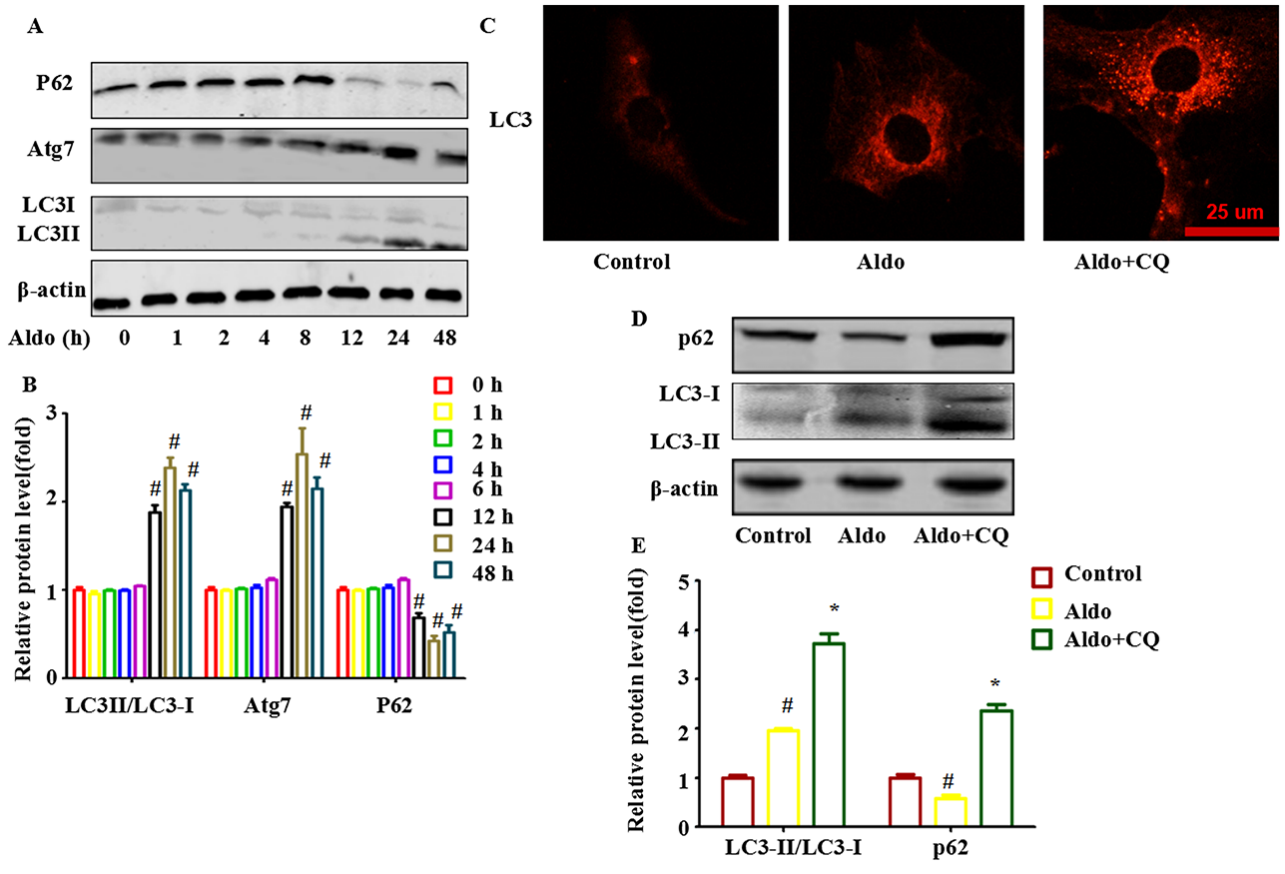
Aldo-induced autophagy flux in cultured podocytes **A.** Western blot analysis revealed the expression of LC3II/LC3I, Atg7, p62, CHOP, Nephrin, Podocin, and β-actin proteins in podocytes after treatment without or with 10-7 M Aldo for various time periods as indicated. **B.** Graphical presentation indicates the relative abundance levels of LC3II/LC3I, Atg7, and p62 after normalization with β-actin. **C.** Immunofluorescence staining for LC3 in podocytes after various treatments, as indicated. The yellow arrow indicates nuclear FOXO1, and the white arrow indicates cytoplasmic FOXO1. Scale bar=25 μm. **D.** Western blot analysis revealed the expression of LC3II/LC3I, p62, and β-actin proteins in podocytes after various treatments, as indicated. **E.** Graphical presentation indicates the relative abundance levels of LC3II/LC3I and p62 after normalization with β-actin. Results (means±SEM) of 3 series of experiments. #P<0.05 vs. normal control, *P<0.05 vs. Aldo alone.

We then aimed to establish whether Aldo affected podocyte autophagy, and if so, at which time points. To analyze autophagy, we examined the formation of LC3-II, the autophagic form of LC3. First, we found that the expression of LC3II /LC3I was up-regulated distinctly as early as 12 h and showed the highest accumulation at 24 h during Aldo stimulation (Figure 2A and 2B). Similar patterns of Atg7 turnover were observed by western blot assay (Figure 2A and 2B). Furthermore, as a selective substrate of autophagy, p62 degradation was also increased by Aldo (Figure 2A and 2B). However, Aldo had no significant effects on Atg5 and Beclin-1 expression (data not shown). Interestingly, autophagy processes (12 h) began earlier than ER stress processes did (24 h) in podocytes stimulated with Aldo.

Furthermore, to monitor autophagic flux, LC3II /LC3I levels were measured in the presence of CQ, which inhibits the acidification of organelles and subsequently autophagosome-lysosome fusion. CQ challenge resulted in further accumulation of LC3II in podocytes after incubation with Aldo (10^−7^ M) for 24 h compared with cells treated with Aldo alone (Figure 2D and 2E). Immunofluorescence staining for LC3 also indicated similar results (Figure 2C). These results suggested that single-treatment with Aldo promoted cellular autophagic flux in podocytes.

### Autophagy attenuated Aldo-induced podocyte injury by improving ER stress in podocytes

**Figure 3:**
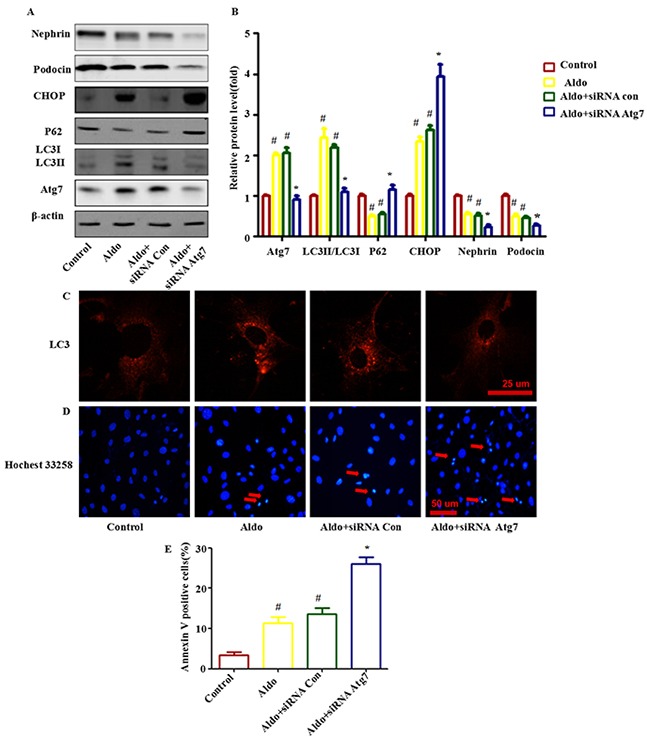
Knocking down Atg7 worsens Aldo-induced ER stress and podocyte injury **A.** Western blot analysis revealed the expression of LC3II/LC3I, Atg7, p62, CHOP, Nephrin, Podocin, and β-actin proteins in podocytes after treatment without or with 10-7M Aldo for various time periods, as indicated. **B.** Graphical presentation indicates the relative abundance levels of LC3II/LC3I, Atg7, p62, CHOP, Nephrin and Podocin after normalization with β-actin. **C.** Immunofluorescence staining for LC3 in podocytes after various treatments, as indicated. Scale bar=25 μm.**D.** Hochest33258 staining in podocytes after various treatments, as indicated. The red arrow indicates apoptosis-induced chromatin condensation and fragmentation. Scale bar=50 μm. **E.** After the various indicated treatments, podocytes were stained with Annexin V and PI and then analyzed by flow cytometry. Quantification of apoptotic cells by flow cytometry. Results (means±SEM) of 3 series of experiments. #P<0.05 vs. normal control, *P<0.05 vs. Aldo alone or Aldo+siRNA Control.

To characterize the role of autophagic activity in Aldo-induced podocyte injury, we inhibited autophagic activity using Atg7-specific siRNA and then assessed Aldo-induced ER stress and podocyte injury markers. Our results suggested that knocking down Atg7 blocked Aldo-induced autophagy processes (Figure 3A through 3C) and worsened Aldo-induced ER stress, as indicated by enhanced CHOP expression (Figure 3A and 3B) and further increased podocyte injury, as evidenced by reductions in the expression levels of the podocyte marker proteins Nephrin and Podocin (Figure 3A and 3B) and the increased number of apoptotic cells compared with podocytes treated with Aldo alone (Figure 3D and 3E).

**Figure 4:**
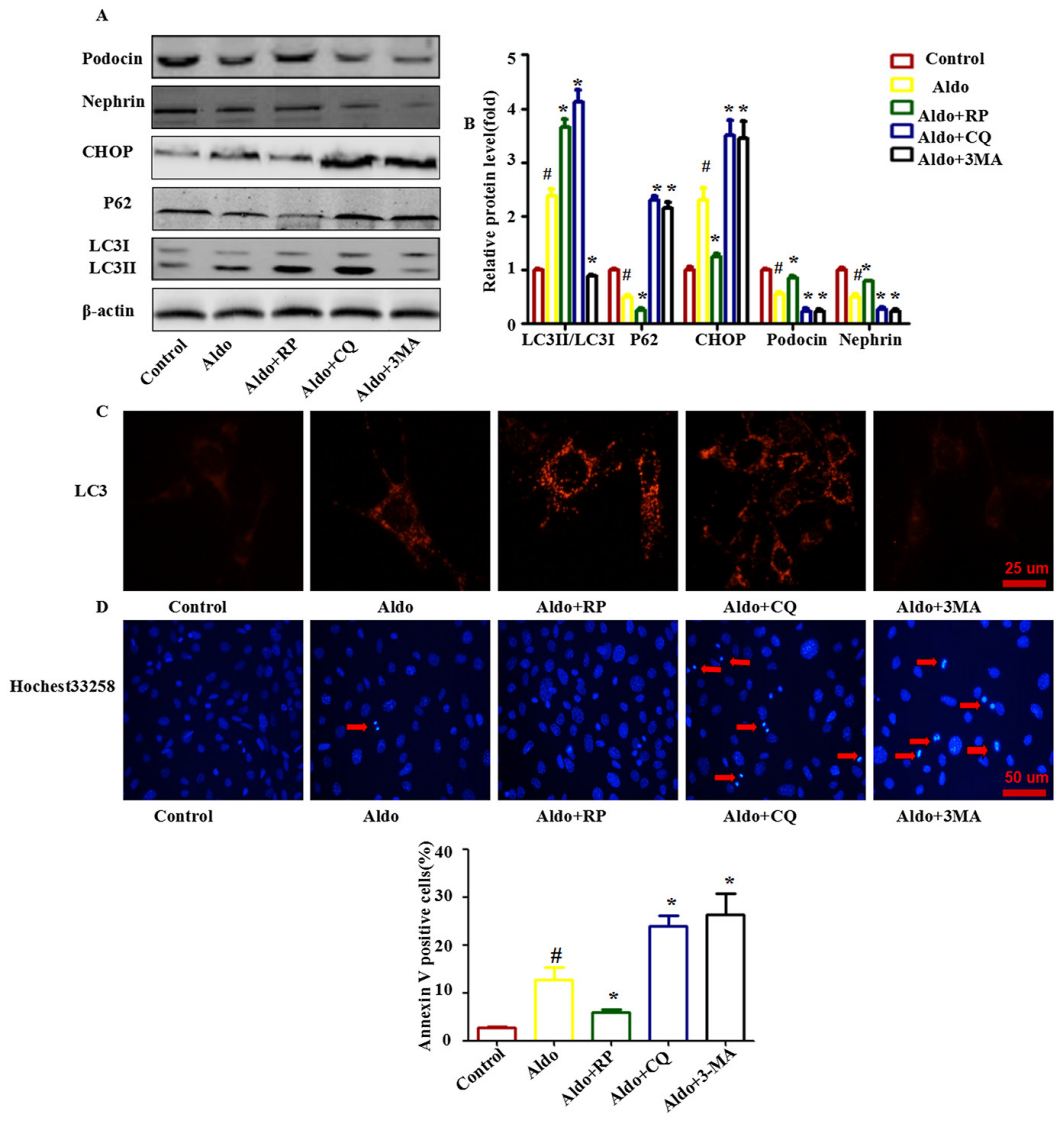
Autophagy protects against Aldo-induced ER stress and podocyte injury **A.** Western blot analysis showed the expression of LC3II/LC3I, p62, CHOP, Nephrin, Podocin and β-actin proteins after treatment with 10-7 M Aldo in the presence or absence of either RP, CQ, or 3-MA for 24 h, as indicated. **B.** Graphical presentation indicates the relative abundance levels of LC3II/LC3I, p62, CHOP, Nephrin, and Podocin after normalization with β-actin. **C.** Immunofluorescence staining for LC3 in podocytes after various treatments, as indicated. Scale bar=25 μm.**D.** Hochest33258 staining in podocytes after various treatments, as indicated. The red arrow indicates apoptosis-induced chromatin condensation and fragmentation. Scale bar=50 μm. **E.** After the various indicated treatments, podocytes were stained with Annexin V and PI and then analyzed by flow cytometry. Quantification of apoptotic cells by flow cytometry. Results (means±SEM) of 3 series of experiments. #P<0.05 vs. normal control, *P<0.05 vs. Aldo alone.

Additionally, treating podocytes with the autophagy inhibitors 3-MA (inhibits the nucleation of autophagosome) or CQ (inhibits the autophagosome-lysosome fusion) also worsened Aldo-induced ER Stress and podocyte injury (Figure 4). Treatment with RP, an inhibitor of mTOR signaling [23], is the most common strategy employed to increase autophagic activity. In contrast, RP treatment increased autophagic activity in podocytes and improved all of the changes (Figure 4). These results demonstrate that autophagy plays a critical protective role in Aldo-induced podocyte injury, possibly by improving overt ER stress.

### Aldo inhibited phosphorylation of Akt but increased mTOR signaling pathway activity

**Figure 5:**
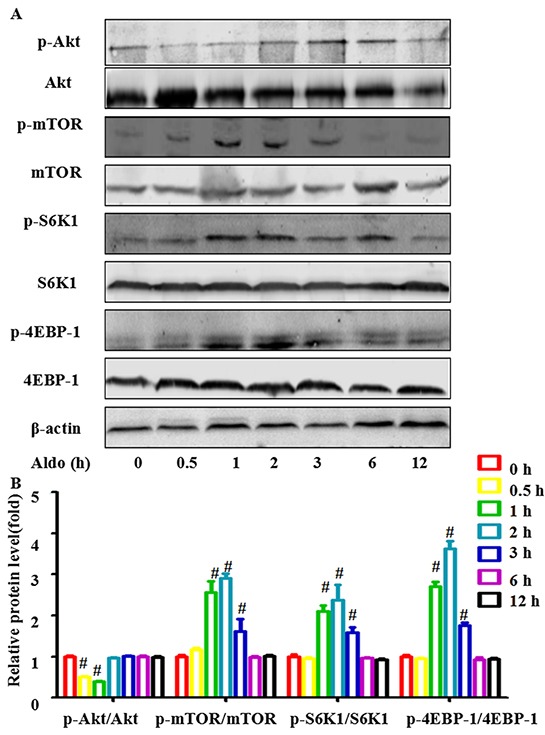
Aldo-induced autophagy is not mediated by inhibiting classic Akt-mTOR signaling in podocytes **A.** Western blot analysis revealed the expression of p-Akt, Akt, p-mTOR, mTOR, p-S6K1, S6K1, p-4EBP1, 4EBP1, and β-actin proteins after treatment with 10-7 M Aldo for various time periods, as indicated. **B.** Graphical presentation shows the relative abundance levels of p-Akt, p-mTOR, p-S6K1, and p-4EBP1 after normalization with Akt, mTOR, S6K1, 4EBP1, and β-actin, respectively. Results (means±SEM) of 3 series of experiments. #P<0.05 vs. normal control.

Inhibition of the Akt-mTOR signaling pathway, a classic method to promote autophagic capacity, plays an important role in maintaining cell homeostasis under various degrees of cellular stress. Hence, we measured whether Akt-mTOR signaling participates in Aldo-induced autophagy. Consistent with a previous study [22], treatment with 10^−7^ M Aldo decreased the level of phosphorylated Akt in a time-dependent manner in the first 120 min; phosphorylation returned to a normal level by 180 min (Figure 5A and 5B). Unexpectedly, mTOR signaling, which is downstream of Akt, was enhanced as evidenced by the increased phosphorylation of mTOR and its two downstream targets, ribosomal protein S6 kinase 1 (S6K1) and eukaryotic initiation factor 4E-binding protein-1 (4EBP-1) [24, 25]. This effect started at 30 min. Maximal stimulation was noted at 120 min and was sustained to 180 min (Figure 5A and 5B). These results suggested that Aldo-induced autophagy did not occur through inhibition of the classic mTOR signaling pathway; thus, other signaling pathways may be involved.

### Aldo-induced FOXO1 expression is required for Aldo-induced compensatory protective autophagy

**Figure 6:**
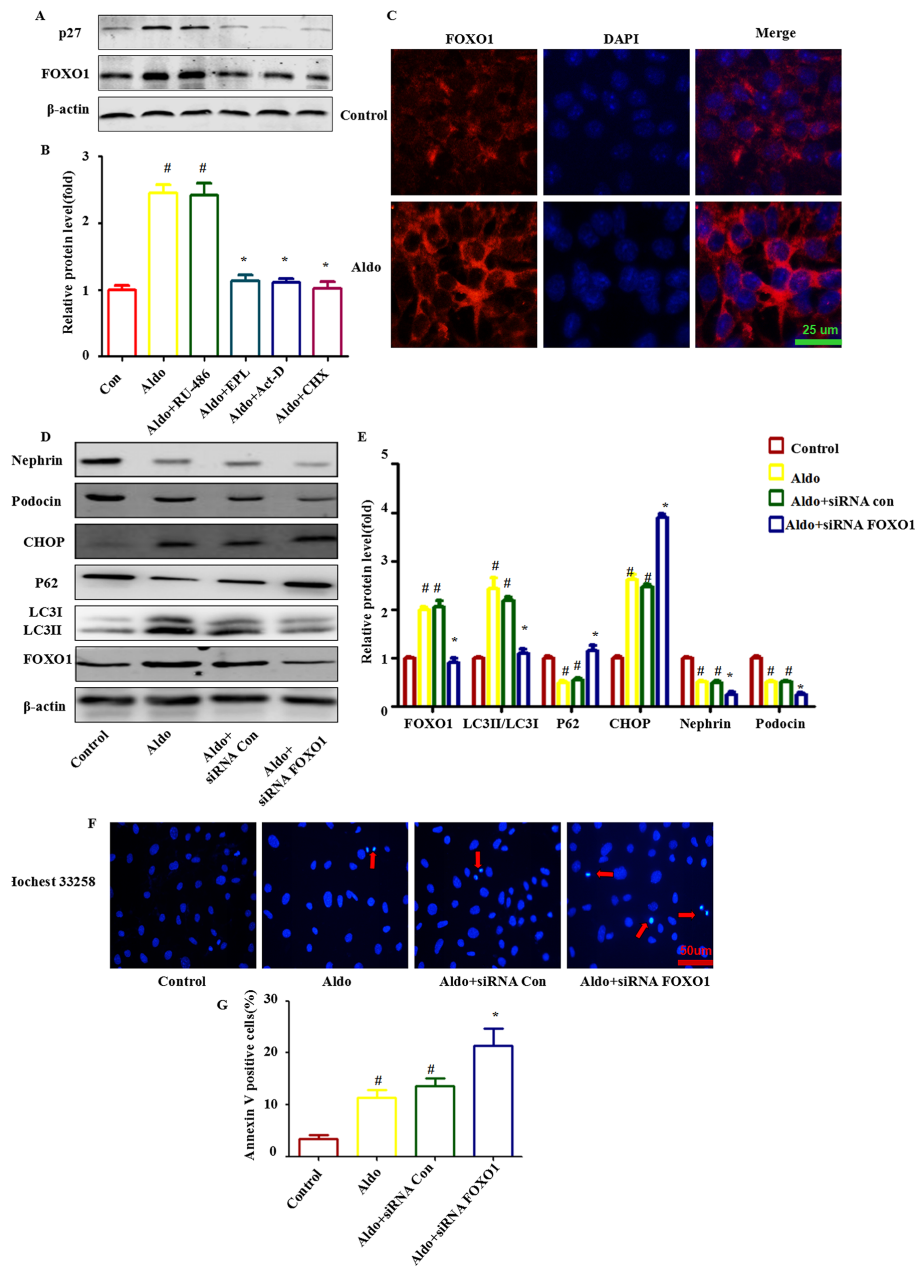
FOXO1 is involved in Aldo-induced compensatory protective autophagy **A.** Western blot analysis revealed the expression of FOXO1, p27, and β-actin proteins after various treatments, as indicated. **B.** Graphical presentation indicates the relative abundances of FOXO and p27 after normalization with β-actin. **C.** Immunofluorescence staining for FOXO1 in podocytes after treatment without or with 10-7M Aldo for 24 h. Scale bar=25 μm. **D.** Equal numbers of podocytes were incubated in media containing buffer (control), siRNA-Con (20 nM) or siRNA-FOXO1 (20 nM) with or without Aldo (10-7 M) for 24 h, as indicated. The whole cell lysate was immunoblotted with antibodies against LC3II/LC3I, p62, FOXO1, CHOP, Nephrin, Podocin, and β-actin. **E.** Graphical presentation indicates the relative abundances of LC3II/LC3I, p62, FOXO1, CHOP, Nephrin and Podocin after normalization with β-actin. **F.** Hochest33258 staining in podocytes after various treatments, as indicated. The red arrow indicates apoptosis-induced chromatin condensation and fragmentation. Scale bar=50 μm. **G.** After the various indicated treatments, podocytes were stained with Annexin V and PI and then analyzed by flow cytometry. Quantification of apoptotic cells by flow cytometry. Results (means±SEM) of 3 series of experiments. #P<0.05 vs. normal control, *P<0.05 vs. Aldo alone or Aldo+siRNA Control.

Because the FOXO family promotes autophagy in skeletal muscle and vascular endothelial cells [26, 27], we hypothesized that FOXO may be responsible for Aldo-induced autophagy in podocytes. FOXO1, a major isoform of the FOXO family in podocytes, was significantly up-regulated following Aldo addition (Figure 6A and 6B). Furthermore, Aldo increased the expression of p27 (a gene directly downstream of FOXO1, Figure 6A and 6B). Given that both the mineralocorticoid receptor (MR) and glucocorticoid receptor (GR) have been reported to mediate the response to Aldo [28], both an MR antagonist (eplerenone, EPL) and a mifepristone antagonist (RU-486) were applied to characterize their roles in Aldo-induced FOXO1 expression. As shown in Figure 6A and 6B, EPL abrogated Aldo-induced FOXO1 expression, but RU-486 did not have this effect. Further, we examined whether this effect was dependent on the non-genomic action of Aldo. However, pretreatment with either actinomycin D or cycloheximide, inhibitors of transcription or protein synthesis, respectively, affected Aldo mediated FOXO1 expression (Figure 6A and 6B). This finding indicates that FOXO1 expression induced by Aldo is dependent upon the initiation of MR-regulated transcriptional events. Additionally, the immunofluorescence results indicated that Aldo induced both FOXO1 expression (Figure 6C).

To further confirm the role of FOXO1 in Aldo-induced podocyte autophagy, FOXO1 siRNA was applied to evaluate their influence on this process. As expected, Aldo triggered an autophagic LC3-II increase and p62 degradation was attenuated by FOXO1 siRNA treatment (Figure 6D and 6E). Additionally, FOXO1 siRNA worsened Aldo induced ER stress and podocyte injury, as evidenced by the increased CHOP expression, reductions in Nephrin and Podocin expression and increased numbers of apoptotic cells (Figure 6D through 6F). This finding suggested that FOXO1 is required for Aldo-induced compensatory protective autophagy.

### FOXO1 increases the expression of Rab7 and FOXO1-induced autophagy is inhibited by Rab7 knockdown

**Figure 7:**
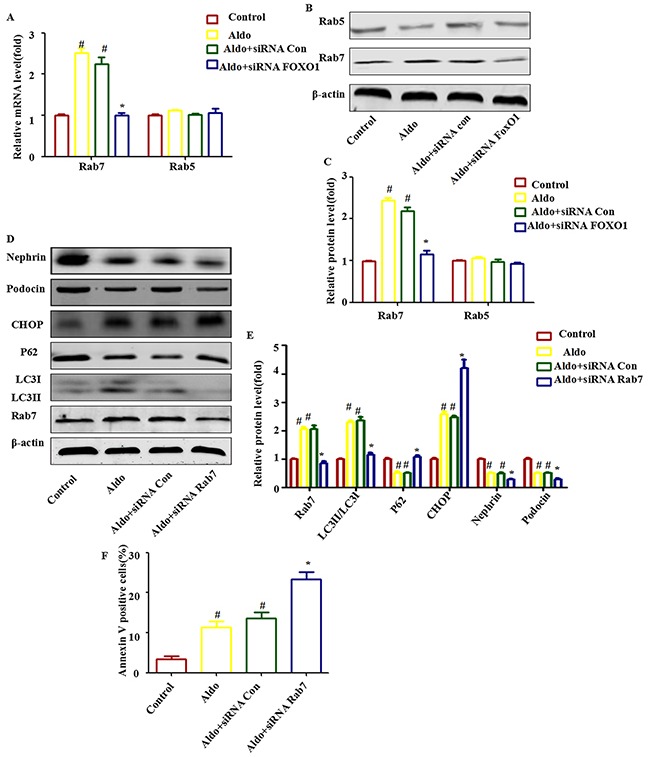
Rab7 participated in FOXO1-mediated autophagy in Aldo-induced podocytes **A.** Real-time RT-PCR analysis revealed the expression of Rab5 and Rab7 mRNAs normalized with GAPDH after various treatments, as indicated. **B.** Equal numbers of podocytes were incubated in media containing buffer (control), siRNA-Con (20 nM) or siRNA-FOXO1 (20 nM) with or without 10-7 M Aldo for 24 h, as indicated. The whole cell lysate was immunoblotted with antibodies against Rab5, Rab7, and β-actin. **C.** Graphical presentation indicates the relative abundance levels of Rab5 and Rab7 after normalization with β-actin. **D.** Equal numbers of podocytes were incubated in media containing buffer (control), siRNA-Con (20 nM) or siRNA-Rab7 (20 nM) with or without 10-7 M Aldo for 24 h, as indicated. **E.** Graphical presentation indicates the relative abundance levels of LC3II/LC3I, p62, Rab7, CHOP, Nephrin, and Podocin after normalization with β-actin. **F.** After the various indicated treatments, podocytes were stained with Annexin V and PI and then analyzed by flow cytometry. Quantification of apoptotic cells by flow cytometry. Results (means±SEM) of 3 series of experiments. #P<0.05 vs. normal control, *P<0.05 vs. Aldo alone or Aldo+siRNA Control.

Rab5 and Rab7 belong to the small GTPase protein family, which stimulates lysosomal biogenesis and the final maturation of late autophagic vacuoles and fusion with lysosomes [29, 30]. Both Rab7 mRNA and protein expression increased significantly during Aldo-induced autophagy in podocytes, but this increase was abolished when FOXO1 was knocked down by FOXO1 siRNA (Figure 7A through 7C). To further evaluate the role of Rab7 in mediating FOXO1-induced autophagy, we treated podocytes with Rab7 siRNA to knockdown Rab7, which was confirmed by immunoblots (Figure 7D and 7E). The FOXO1-induced increase in LC3-II/LC-I expression and p62 degradation was significantly attenuated by knockdown of Rab7 (Figure 7D and 7E). As expected, Rab7 siRNA treatment worsened Aldo-induced ER stress and podocyte injury, as evidenced by the reductions in Nephrin and Podocin expression and the increased numbers of apoptotic cells (Figure 7E and 7F). These results suggest that stimulation of autophagic flux by Aldo in podocytes is mediated through up-regulation of the FOXO1/Rab7 axis. However, minimal change in Rab5 expression was noted (Figure 7A through 7C).

### Aldo increased the level of FOXO1 acetylation through P300 in cultured podocytes

**Figure 8:**
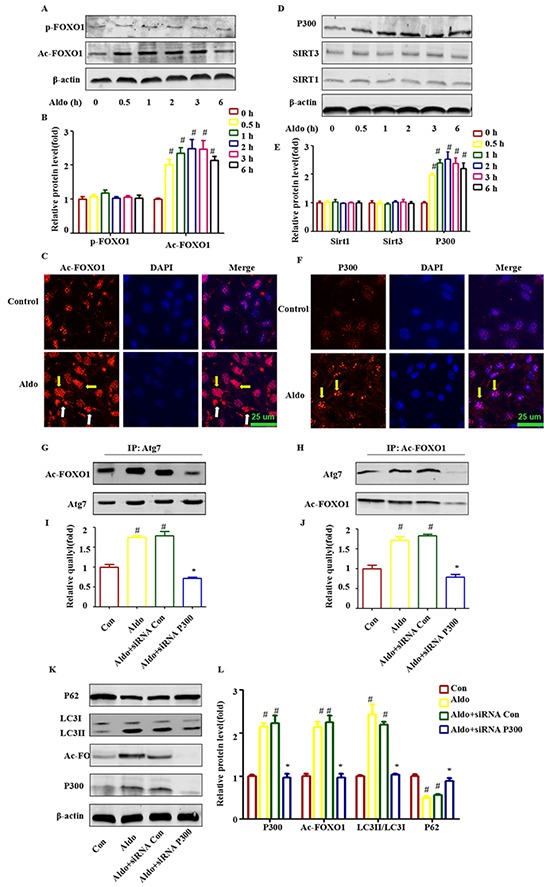
Post-translational modification of FOXO1 is also responsible for Aldo-induced autophagy in cultured podocytes **A, D.** Western blot analysis indicated the expression of Ac-FOXO1, p-FOXO1, P300, Sirt1, Sirt3, and β-actin proteins in podocytes after treatment with or without 10-7 M Aldo for various time periods, as indicated. **B, E.** Graphical presentation shows the relative abundance levels of Ac-FOXO1, p-FOXO1, P300, Sirt1, and Sirt3 after normalization with β-actin. **C, F.** Immunofluorescence staining for Ac-FOXO1 and P300 in podocytes after treatment without or with 10-7M Aldo for 4 h. The yellow arrow indicates nuclear Ac-FOXO1 or P300, and the white arrow indicates cytoplasmic Ac-FOXO1. Scale bar=25 μm.**G, H.** Co-immunoprecipitation of Atg7 and Ac-FOXO1 in podocytes. Cells were treated as indicated, and the cell lysate was then extracted for co-immunoprecipitation with anti-Ac-FOXO1or Atg7 followed by probing with anti-acetylated Atg7 or Ac-FOXO1 (n=3). **I, J.** Graphical presentation shows the relative quality of Atg7 and Ac-FOXO1 after normalization with Ac-FOXO1 or Atg7 respectively. **K.** Equal numbers of podocytes were incubated in media containing buffer (control), siRNA-Con (20 nM) or siRNA-P300 (20 nM) with or without 10-7 M Aldo for 24 h, as indicated. **L.** Graphical presentation shows the relative abundance levels of P300, Ac-FOXO1, LC3II/LC3I, and p62 after normalization with β-actin. Data are presented as the means±SEM of 3 independent experiments. #P<0.05 vs. normal control, *P<0.05 vs. Aldo alone or Aldo+siRNA Control.

Accumulating evidence has demonstrated that a post-translational modification of cytosolic FOXO1 was also required for triggering autophagy in cells [19, 20, 26]. As shown in Figure 8A and 8B, acetylation of FOXO1 (Ac-FOXO1) was increased time dependently under the influence of 10^−7^ M Aldo, and Ac-FOXO1 began to exhibit enhancement as early as 30 min. However, no significant change in FOXO1 phosphorylation was noted (Figure 8A and 8B). Interestingly, Aldo induced both cytoplasmic and nuclear AC-FOXO1 expression (Figure 8C). Next, we searched for evidence of any down-regulation of histone deacetylase (HDAC) or up-regulation of histone acetylase in podocytes during Aldo treatment. As shown in Figure 8D and 8E, no significant changes in the expression levels of histone deacetylases, including Sirt1 and Sirt3 (a NAD+-dependent histone deacetylase family), were noted. In addition, we did not detect Sirt2 expression in cultured podocytes (data not shown). However, Aldo increased histone acetylase (P300) as early as 4 h after treatment with 10^−7^ M Aldo, which is consistent with the change in Ac-FOXO1. Immunofluorescence staining results indicated P300 expression increased mainly in the nucleus (Figure 8F).

We previously mentioned that Aldo treatment increased Atg7 expression (Figure 1A and 1B), and previous reports have demonstrated that promoting the interaction of Ac-FOXO1 and Atg7 could accelerate the process of autophagy [20, 26]. We further evaluated whether the interaction between Atg7 and Ac-FOXO1 was also involved in the induction of the autophagic process in podocytes stimulated by Aldo. As expected, Aldo increased the interaction of Ac-FOXO1 and Atg7 as early as at 4 h after stimulation by 10^−7^ M Aldo (Figure 8G through 8J) Additionally, P300 siRNA not only significantly inhibited the Ac-FOXO1 level but also the interaction between Atg7 and Ac-FOXO1 (Figure 8G through 8J), which is accompanied by the inhibition of autophagy, as indicated by decreased LC3-II and increased p62 accumulation (Figure 8K and 8L). These results suggested that P300 was responsible for the acetylation of FOXO1 and that the P300/FOXO1 axis participated in the regulation of the autophagic process in podocytes stimulated by Aldo.

### Defective autophagy in Aldo-induced kidney injury in an Aldo/salt-induced model

To gain *in vivo* support for the protective effect of autophagy on podocytes, we used a rat model with Aldo infusion for 28 days in the absence and presence of RP or CQ. All physiologic and biochemical data are presented in Table 1. Body weights were significantly lower in rats treated with RP or CQ compared with control or Aldo/salt-treated rats. Aldo infusion significantly increased the kidney/body weight ratio, urine volume, and urinary protein/creatinine ratio compared with control rats. In the Aldo/RP group, the urine volume and urinary protein/creatinine ratio were significantly reduced compared with the Aldo/salt group. However, treatment with RP had no effect on the kidney/body weight ratio compared with Aldo/salt-treated animals. In the Aldo/CQ group, urine volume and the kidney/body weight ratio were significantly reduced, whereas the urinary protein/creatinine ratio was further significantly increased compared with Aldo/salt-treated animals. Urinary Aldo levels were increased in all Aldo groups, accounting for correct pump function. No significant differences in serum creatinine levels were noted among Aldo-infused groups. Systolic blood pressure (SBP) did not increase in control animals over 28 days (final measurement, 140±3 mm Hg). Aldo/salt treatment resulted in marginally increased SBP over time (final measurement, 188±5 mmHg). Administration of RP had no effect on SBP compared with Aldo/salt-treated rats (final measurement, 191±6 mmHg). Interestingly, administration of CQ in Aldo/salt-treated rats resulted in reduced SBP (161±5 mmHg).

**Table 1:** Biological parameters of rats in the control, Aldo/salt, Aldo/RP, and Aldo/CQ group, respectively, at 4 weeks

	Control (n=6)	Aldo/salt (n=6)	Aldo/RP (n=6)	Aldo/CQ (n=6)
Body weight (g)	464±11	452±9	305±7*	351±6*
Kidney weight/body weight ratio (mg/g)	5.8±0.2	9.5±0.6*	9.1±0.6*	7.6±0.3*,**
Urine volume (ml)	12±3	45±11*	33±5*,**	15±4**
Creatinine clearance (ml/min)	4.1±0.4	2.9±0.2	4.5±0.6	2.8±0.3
Albumin/creatinine (mg/mg)	1.4±0.5	9.3±2.4*	5.7±1.4*,**	11.2±3.0*,**
Urinary aldosterone at end (μ/24 h)	0.04±0.01	0.15±0.03*	0.14±0.01*	0.14±0.02*
SBP (mmHg)	140±3	188±5*	191±6*	161±5*,**

* P<0.05 vs. control values

** P<0.05 vs Aldo treatment

**Figure 9:**
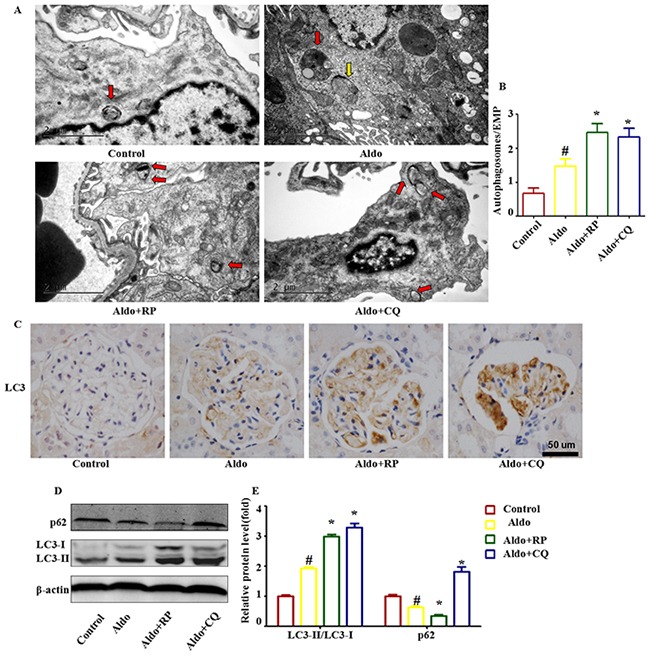
Effects of RP and CQ on autophagy in Aldo-induced rats **A.** Representative electron micrographs showing autophagic vacuoles in podocytes. The red arrow indicates autophagosomes, and the yellow arrow indicates autolysosomes. Scale bar=2 μm. **B.** Graphical presentation indicates the abundance of autophagic vacuoles in various groups, as indicated. **C.** Immunohistochemical staining for LC3 in rat kidney tissue from various groups, as indicated. Scale bar=50 μm. **D.** Western blot analysis revealed the expression of LC3II/LC3I, p62, and β-actin proteins after various treatments in rats, as indicated. **E.** Graphical presentation shows the relative abundance levels of LC3II/LC3I and p62 after normalization with β-actin. Results (means±SEM) of 3 series of experiments. #P<0.05 vs. normal control, *P<0.05 vs. Aldo alone.

We then confirmed the occurrence of autophagy in podocytes after Aldo/salt administration in SD rats. By electron microscopy, we detected the formation of autophagic vacuoles in podocytes during Aldo/salt treatment in rats (Figure 9A and 9B). Morphologically, the formation of autophagosomes in kidneys was visualized by immunohistochemical staining of LC3. Four weeks of Aldo/salts treatment led to a notable increase of LC3-II in glomeruli, indicating the formation of autophagosomes (Figure 9C). Western blot assays also revealed a significant increase in the LC3-II protein level and a decrease in the p62 level in the glomeruli of model rats compared with the control rats (Figure 9D and 9E). These results demonstrated that Aldo induced autophagy in podocytes *in vivo*. Administration of CQ or RP induced a similar effect on LC3-II and p62 as observed in cell-based experiments (Figure 9B through D). These data indicate that Aldo induced an autophagic response, which could be modulated by RP and CQ in model rats.

**Figure 10:**
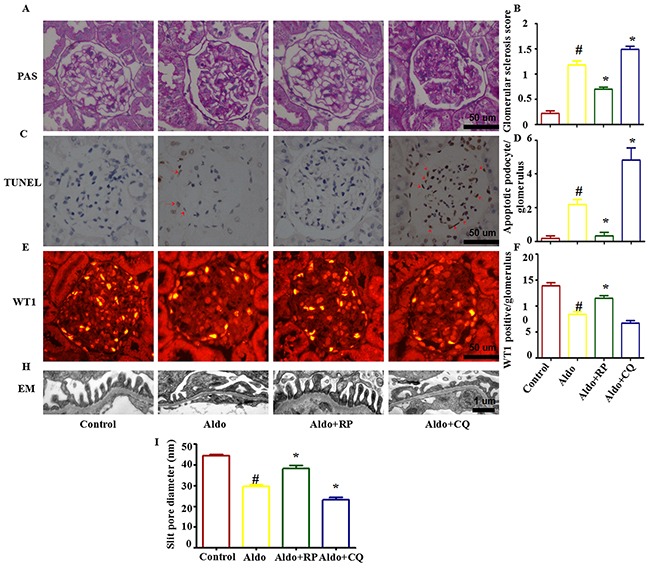
Effects of RP and CQ on podocyte injury in Aldo-induced rats **A.** The light microscopic appearance of representative glomeruli stained with PAS. Scale bar=50 μm. **B.** Results of the semi-quantitative analysis. **C.** The TUNEL assay in renal cortex sections of control and Aldo-infused rats. The red arrow indicates TUNEL-positive podocytes. Scale bar=50 μm.**D.** The bar graph indicates the mean number of TUNEL-positive podocytes per glomerular cross-section. **E.** Representative images of WT-1 immunostaining, a surrogate marker for podocyte number, in kidney tissues from each group mentioned above. Scale bar=50 μm.**F.** Quantification of WT-1-positive cells per glomerulus. **G.** Foot processes of podocytes by transmission electron microscopy (TEM) Scale bar=1 μm. **H.** Quantitative analysis of the slit pore diameter. Results (means±SEM) of 6 series of experiments. *P<0.05 vs. normal control, #P<0.05 vs. Aldo alone.

**Figure 11:**
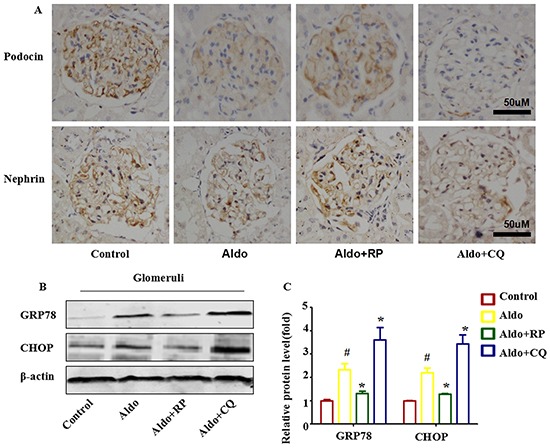
Autophagy protects podocytes against Aldo-induced ER stress and podocyte injury in vivo **A.** Immunohistochemical staining for nephrin and podocin in rat kidney tissue from various groups, as indicated (n=6). Scale bar=50 μm.**B.** Western blot analysis showed the expression of CHOP, GRP78, and β-actin proteins in the glomeruli of rats from various groups, as indicated. **C.** Graphical presentation shows the relative abundance levels of CHOP and GRP78 and after normalization with β-actin (n=3). #P<0.05 vs. normal control, *P<0.05 vs. Aldo alone.

In the presence of CQ, the severity of the glomerular injury induced by Aldo was further increased, revealing glomerular enlargement and increased mesangial area (Figure 10A). Semi-quantification confirmed that CQ significantly further increased glomerular damage during Aldo treatment (Figure 10B). Further examination of renal tissues by TUNEL assay indicated that Aldo induced podocyte apoptosis, which was further increased by CQ treatment (Figure 10C and 10D). Given the increase in TUNEL-positive cells in CQ-treated rats, immunostaining for Wilms's tumor protein (WT-1), a surrogate marker for podocyte number, was performed to determine if the decrease in WT-1-positive cells was secondary to apoptosis. Compared with the Aldo group (13.6±2.3), the number of podocytes was further decreased in CQ- combined with Aldo-treated rats (8.2±1.1); however, no significant difference was noted between these two groups (Figure 10E and 10F). In the electron micrographs, extensive fusion of foot processes was more severe, and the slit pore diameter (Figure 10G and 10H) and the Nephrin and Podocin expression levels were further decreased in CQ-treated rats compared with the Aldo infusion group (Figure 11A). Additionally, the expression levels of the ER stress markers CHOP and GRP78 were further increased in CQ-treated rats compared with the Aldo infusion group (Figure 11B and 11C). In contrast, treatment of the Aldo-infused rats with RP activated an autophagy flux and protected against Aldo-induced podocyte injury by attenuating ER stress (Figure 10 and 11). Collectively, these results demonstrate a renoprotective role of autophagy in this disease model.

### FOXO1, but not the mTOR signaling pathway, is responsible for Aldo-induced autophagy in the glomeruli of rats

**Figure 12:**
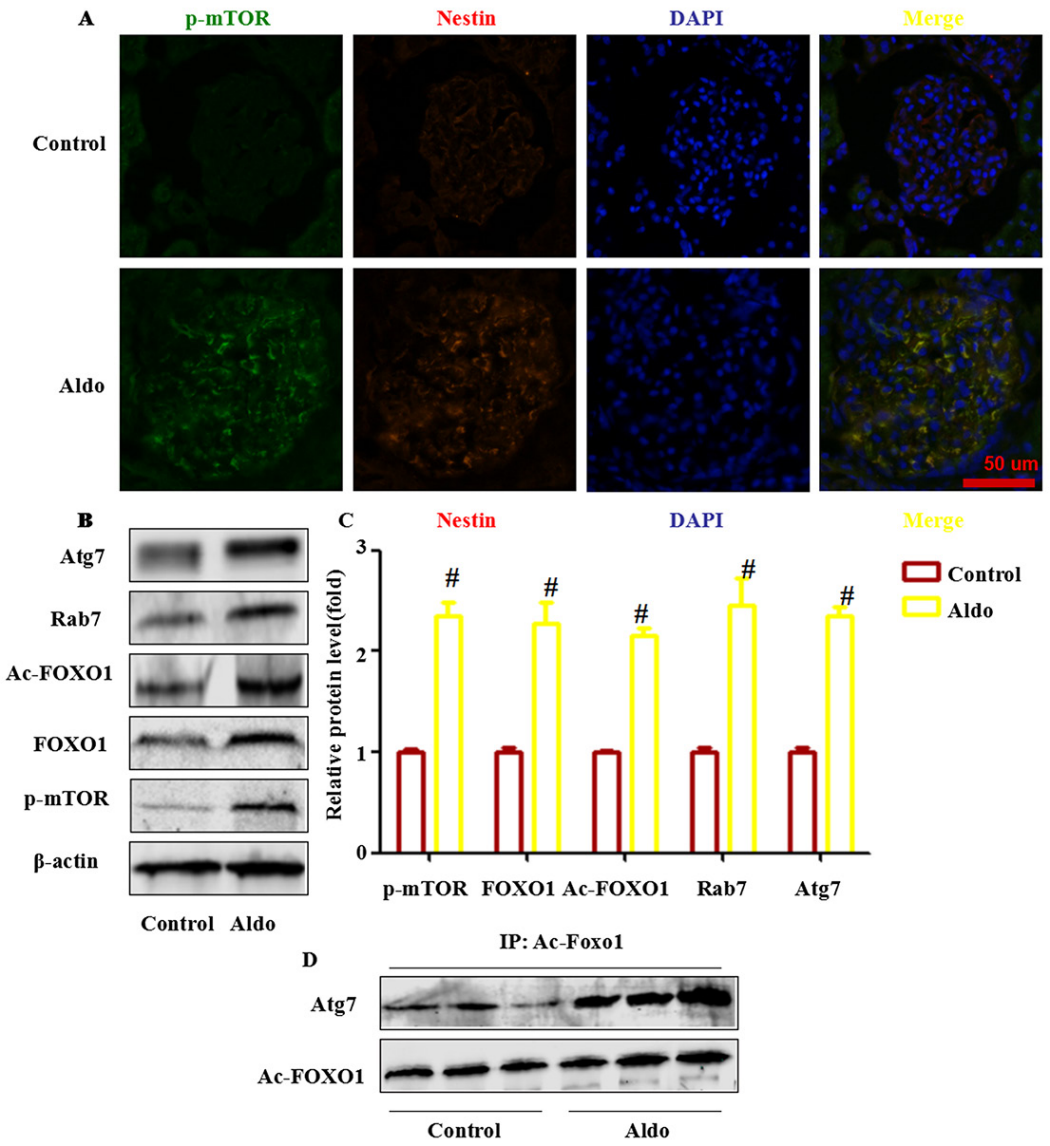
FOXO1 and acetylation of FOXO1 is associated with Aldo-induced podocyte autophagy in vivo **A.** Representative images demonstrating the expression and localization of p-mTOR (green), nestin (red), and DAPI (blue) by indirect immunofluorescent staining in frozen sections of kidneys from rats in each group, as indicated. Scale bar=50 μm.**B, C.** Western blot analysis revealed the expression of p-mTOR, FOXO1, Ac-FOXO1, Atg7, Rab7 and β-actin proteins from isolated glomeruli from control and Aldo-treated rat kidneys. **D.** Co-immunoprecipitation of Atg7 and Ac-FOXO1 in glomeruli. Glomeruli were isolated from the fresh kidney tissue in two groups, as indicated. Then, the tissue lysate was extracted for co-immunoprecipitation with anti- Ac-FOXO1 followed by probing with anti-acetylated Atg7. Results (means±SEM) of 3 series of experiments. #P<0.05 vs. normal control.

We next examined whether Aldo activated mTOR signaling in the kidneys of Aldo-induced rats the same as demonstrated in cultured podocytes. Morphologically, 4 weeks of Aldo/salts treatment led to a notable increase of p- mTOR in glomeruli, indicating the activation of mTOR signaling. Co-immunostaining with nestin, a marker of podocytes, indicated that most of the p- mTOR was localized in the podocytes (Figure 12A). Western blot assays also exhibited a significant increase in the p- mTOR protein levels in the glomeruli of model rats compared with control rats (Figure 12B and 12C).

Based on the results of cell-based experiments and our previous animal experimental results that indicate that Aldo infusion also induced podocyte autophagy *in vivo*, we hypothesized that Aldo-induced autophagy in the glomeruli of rats may be associated with increased FOXO1, ac- FOXO1, Rab7, and the interaction between ac-FOXO1 and Atg7. As expected, western blot assays revealed that Aldo infused rats increased FOXO1, Rab7, ac-FOXO1, and Atg7 expression (Figure 12B and 12C). The IP assay indicated that Aldo enhanced the interaction of ac-FOXO1 and Atg7 (Figure 12D).

## DISCUSSION

**Figure 13:**
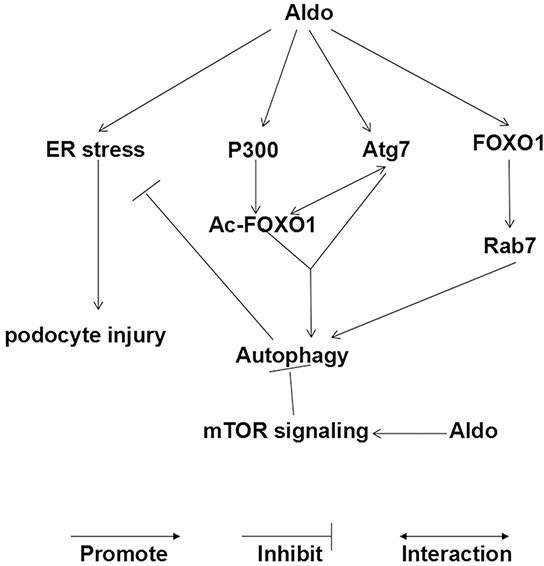
Working model of Aldo-induced compensatory autophagy in podocytes (i) Aldo activates mTOR signaling, which inhibits autophagy in podocytes. (ii) However, Aldo simultaneously controls the following aspects to promote the autophagic process: Aldo induced the expression of FOXO1 and its downstream protein (Rab7); Aldo enhanced the acetylation of FOXO1 through P300 and increased the interaction between Ac-FOXO1 and Atg7. The balance between the effect of Aldo on autophagy in our model favors FOXO1 signaling, which can override the potential inhibitory effect of activated mTOR signaling. (iii) ER stress is involved in Aldo-induced podocyte injury, and autophagy protected podocytes against the injury by attenuating ER stress, serving as a compensatory protective mechanism.

In the present study, we provide *in vitro* and *in vivo* evidence that Aldo stimulated autophagic flux, a compensatory protective mechanism to reduce podocyte injury by inhibiting ER stress. Additionally, Aldo-induced adaptive autophagy is mediated through the up-regulation of the FOXO1/Rab7 axis and the post-translational modification of FOXO1 rather than inhibiting the classic Akt-mTOR pathway (Figure 13).

Evidence for impaired autophagic flux *in vivo* is emerging in various other animal models of CKD, including minimal change nephrotic syndrome [31], diabetic nephropathy [32], ischemia-reperfusion injury [33], and focal segmental glomerulosclerosis (FSGS) [34]. Consistent with a previous study [16], autophagy flux is induced rather than inhibited in an Aldo-induced podocyte injury model (Figures 2 and 8).

Increasing autophagy attenuated Aldo-induced podocyte injury, whereas inhibiting autophagy worsened Aldo-induced podocyte injury both in vivo and in vitro (Figure 3, 4 and 9). These results indicated that Aldo-induced autophagy in podocytes is a self-protective mechanism to relieve stress-related podocyte injury. An interesting finding was that RP impaired weight gain in Aldo-infused rats, which is consistent with previous results that rats treated with RP had low body weight [23, 35]. S6K1, downstream of mTOR, protects against age- and diet-induced obesity in mice [36]. Furthermore, single intraperitoneal RP led to decreased food intake and daily weight gain [37], both of which may offer a potential explanation for the consistent inhibitory effect of RP on weight gain. Animals that were administered CQ also lost weight throughout the 4-week period with a concomitant reduction in food consumed (data not shown), which is consistent with a previous study [38]. Reduction in blood pressure associated with long-term CQ treatment has been previously reported in rats [39] and humans [40]. The low mean arterial blood pressure following CQ administration, presumably leading to a reduction of glomerular filtration rate (GFR), may contribute to the decrease in urine volume (Table 1).

Consistent with previous studies [16, 41, 42], ER stress contributes to glomerular and tubular damage in kidney disease: we found that the expression levels of the ER stress-associated proteins, GRP78 and CHOP, were up-regulated following Aldo stimulation both *in vivo* and *in vitro*. Additionally, increasing autophagy attenuated Aldo-induced ER stress, whereas inhibiting autophagy aggravated ER stress both *in vivo* and *in vitro* (Figure 3, 4 and 11). Interestingly, Aldo induced compensatory autophagy as early as 12 h following treatment, which is earlier than the induction of ER stress (24 h) in cultured podocytes (Figure 2A and 2B). This finding emphasizes the dominating role of autophagy in podocyte adaptation to stress, which can override the potential toxicity of overt ER stress at early times. However, if the cell stress is consistent or irreversible, cell injury ensues.

mTOR signaling is a master negative regulator of autophagy. Surprisingly, mTOR signaling is activated after stimulation with Aldo both *in vitro* (Figure 5A and 5B) and *in vivo* (Figure 12A through 12C), indicating that an additional mTOR-independent mechanism must be involved in Aldo-induced autophagy. Although Aldo-induced podocyte autophagy is not mediated by inhibiting mTOR signaling, RP treatment could still further increase podocyte autophagy induced by Aldo. This means that Aldo could increase autophagy flux more robustly if the negative regulatory effect of the increased mTOR signaling is absent. Interestingly, chronic mTOR inhibition disrupts the autophagic pathway in podocytes *in vitro* [43], and it has yet to be discovered at which dose and how long treatment with RP is detrimental or beneficial to promote podocyte autophagy flux *in vivo*. Nonetheless, RP is obviously unsuitable for CKD treatment due to its multiple side effects [44, 45] and its contradictory effect on autophagy flux in podocytes.

Regulation of the expression and transcriptional activity of FOXO1 is involved in promoting cellular autophagy [26, 31, 46]. In our study, Aldo up-regulated the expression of FOXO1 (Figure 6A through 6C) and its downstream effector Rab7 (Figure 7A through 7C), which contributes to the transport of late endosomes and lysosomes in the autophagic-endocytic pathway [47]. Consistently, previous studies have demonstrated that Rab7 plays an important role in mediating FOXO1-induced stimulation of autophagy, [26, 31] whereas our results indicate that Rab7 was enhanced following the treatment of Aldo (Figure 7A through 7C, Figure 12B and 12C). Rab7 knockdown significantly attenuated FOXO1-mediated protective autophagy in Aldo stimulated podocytes (Figure 7D through 7F). Thus, we conclude that Aldo accelerates the podocyte autophagic process at least partially by enhancing the FOXO1/Rab7 axis.

Posttranslational modifications including phosphorylation and acetylation of FOXO1 were reported to participate in the regulation of autophagy [20, 31]. According to our findings, Aldo induced P300 expression but had no effect on Sirt1 or Sirt3 expression, which suggests that the Aldo-induced acetylation of FOXO1 occurs by increasing P300 expression rather than by reducing the expression levels of Sirt family members (Figure 8A through 8E). Interestingly, Aldo-induced P300 is located in the nucleus (Figure 8F); however, Aldo-enhanced Ac-FOXO1 was located in both in the nucleus (major) and the cytoplasm (minor) (Figure 8C), suggesting that Ac-FOXO1 transferred from the nucleus to the cytoplasm. However, no significant change in the phosphorylation of FOXO1 was noted (Figure 8A and 8B). Acetylation promotes the phosphorylation of FOXO1 via the mTOR signaling pathway [48], thus explaining why no obvious changes in p-FOXO1 and activated mTOR signaling were noted. This effect should have been attenuated by the negative regulation of activated Akt (Ser 473) induced by Aldo. As expected, the interaction between Ac-FOXO1 and Atg7, which has also been confirmed to promote autophagy, was also found in both Aldo-induced cultured podocytes (Figure 8G through 8J) and a rat model (Figure 10D). Furthermore, P300 knockdown by special siRNA significantly attenuated Aldo-induced acetylation of FOXO1 and the interaction between Ac-FOXO1 and Atg7 and was accompanied with reduced autophagy compared with podocytes treated with Aldo alone (Figure 7K and 7L). Hence, these results suggest that Aldo accelerates the podocyte autophagic process that was also partially mediated by enhancing the acetylation of FOXO1 and its interaction with Atg7.

The striking finding of this study is the proof of concept that autophagy is a key adaptive mechanism in Aldo-induced podocyte injury. In addition, we are the first to identify that FOXO1 could be a potential therapeutic target to improve podocyte injury through the promotion of autophagy in podocytes.

## MATERIALS AND METHODS

### Antibodies and reagents

Aldo, rapamycin (RP), chloroquine (CQ), 3-methyladenine (3-MA), tunicamycin (Tun), tauroursodeoxycholic acid (TAUDC), and anti-β-actin antibody were purchased from Sigma (St Louis, MO). Antibodies against LC3, Akt, p-Akt, mTOR, p- mTOR, S6K1, p-S6K1, 4EBP1, p-4EBP1, GRP78, GRP94, CHOP, FOXO1, p-FOXO1, Rab5, and Rab7 were purchased from Cell Signaling Technology (Beverly, MA). Anti-Podocin, anti-Nephrin, and anti-p62 antibodies were obtained from Abcam (Cambridge, MA). P300, Ac-FOXO1, and nestin antibodies were obtained from Santa Cruz Biotechnology, Inc. (Santa Cruz, CA).

### Podocyte culture and treatment

The MPC5 conditionally immortalized mouse podocyte cell line was cultured as previously described [5]. Podocytes were maintained without interferon-γ at 37°C for 14 days before experimentation to induce differentiation. Differentiated podocytes were made quiescent in medium that contained 0.1% fetal bovine serum (FBS) for 24 h, and the cells were then exposed to treatment for the indicated time periods.

### Transient transfection of cells with siRNA

For knockdown experiments, podocyte cells were transiently transfected with siRNA specifically targeting P300, FOXO1, Rab7 or a negative control siRNA, respectively, using Lipofectamine 3000 (Invitrogen, Carlsbad, CA) according to the manufacturer's instructions. Cells were transfected with 20 nM CHOP, P300, FOXO1, Rab7 or control siRNA for 24 h before further treatment. Cellular protein was extracted and subjected to western blot analysis for detection of CHOP, P300, FOXO1, and Rab7.

### Hoechst 33258 staining

Podocytes grown on glass cover slips in the different groups were stained with Hoechst 33258 and viewed by fluorescence microscopy for the measurement of apoptosis.

### Annexin V-fluorescein isothiocyanate conjugated with propidium iodide (PI) staining

After treatment, podocytes from different groups were quantified by Annexin V/PI staining according to the manufacturer's instructions (BD Biosciences, San Diego, CA) as described previously [6]. Briefly, podocytes (10^6^ cells/ml) were harvested and centrifuged at 1000 rpm for 5 min. After washing twice with phosphate-buffered saline (PBS), the cells were resuspended in 500 μl of ice-cold binding buffer and then incubated with 2 μl of Annexin V and 2 μl of PI for 15 min in the dark. After resuspension in 200 μl of binding buffer, the cells were analyzed on a FACScan flow cytometer (Epics Altra, Beckman Coulter, Brea, CA).

### Immunofluorescence staining

Indirect immunofluorescence staining was performed according to an established procedure. Briefly, the cells were fixed with cold acetone for 10 min, and cryosections of 5 μm thickness were prepared. After being blocked with 3% bovine serum albumin (BSA) for 1 h, the cells and sections were incubated overnight at 4°C with primary antibodies against LC3 (1:200), P300 (1:200), p-mTOR (1:100), and nestin (1:200) in PBS containing 3% BSA. Sections were then washed with PBS and incubated in the dark with fluorescein isothiocyanate (FITC)- and tetramethylrhodamine isothiocyanate (TRITC)-conjugated secondary antibodies (Sigma-Aldrich) at a 1:200 dilution in PBS containing 3% BSA for 1 h. After being thoroughly washed with PBS, slides were mounted with 4′,6-diamidino-2-phenylindole (DAPI, Vector Laboratories, H-1200) and viewed with a Nikon Eclipse 80i Epi-fluorescence microscope equipped with a digital camera (DS-Ri1, Nikon).

### Real time-polymerase chain reaction (PCR)

Real time-PCR was performed with THUNDERBIRD SYBR qPCR Mix reagent (TOYOBO, QPS-201) in a real-time PCR system (Stratagene). The following primer pairs were used: RAB7 (sense 5‘-GGGGA CTCTGGTGTTGGAA-3′; antisense 5‘-CGCTCCTATTG TGGCTTTGT-3′); RAB5 (sense 5‘-AGCCAGAAGCCAG TGTTGTA-3′; antisense 5‘-GGTTTTTGCCATTCAGGAA GA-3′; and GAPDH (sense 5‘-TCTTTTGCGTCGCCAGCC GAG-3′; antisense 5‘-TCC CGTTCTCAGCCTTGAC GGT-3′).

### Western blot analysis and immunoprecipitation (IP) assay

Podocytes harvested from plates and sieved glomeruli were lysed in sodium dodecyl sulfate (SDS) sample buffer containing 150 mM NaCl, 0.1% Triton X-100, 0.5% deoxycholate, 0.1% SDS, 50 mM Tris–HCl (pH 7.0), and 1 mM ethylenediaminetetraacetic acid (EDTA). Detection of protein expression by western blot was performed according to established protocols. Equal amounts of protein were subjected to SDS- polyacrylamide gel electrophoresis (PAGE) on 10 to 12% polyacrylamide gels and transferred to a polyvinylidene difluoride membrane (Millipore, HATF09025). The membrane with blotted protein was blocked for 1 h with blocking buffer containing 5% nonfat dry milk and 0.05% Tween-20 in Tris-buffered saline (TBS-T), followed by incubation with the primary antibodies as follows: LC3 (1:1000), p62 (1:2000), Nephrin(1:1000), Podocin (1:1000), Akt (1:1000), p-Akt (1:1000), mTOR (1:1000), p-mTOR (1:1000), S6K1 (1:1000), p-S6K1 (1:1000), p4EBP1 (1:1000), p-p4EBP1 (1:1000), GRP78 (1:1000), GRP94 (1:1000), CHOP (1:1000), P300 (1:200), FOXO1 (1:1000), Ac-FOXO1 (1:200), p-FOXO1 (1:1000), Rab5 (1:1000), Rab7 (1:1000) and β-actin (1:5000). Then, the membrane was washed thrice with TBS-T for 30 min and incubated at room temperature for 1 h with diluted (1:10, 000) secondary horseradish peroxidase-conjugated goat anti-rabbit IgG or goat anti-mouse IgG. Whole-cell and freshly isolated glomeruli lysate proteins were used for immunoprecipitation (IP) with Ac-FOXO1 or Atg7. One milligram of antibody was added to 1 ml of the lysate, which was incubated at 4°C overnight. After the addition of Protein A/G-agarose beads, the incubation was continued at 4°C overnight. Immunoprecipitates were extensively washed with lysis buffer and eluted with SDS loading buffer by boiling for 5 min. The bands were detected using the ChemiDoc XRS System (Bio-Rad). The relative intensity of each band was normalized to β-actin.

### Animals

Study protocols were reviewed and approved by the Institutional Animal Care and Use Committee at Fudan University, China. In brief, 24 male Sprague-Dawley rats (5 to 6 weeks old, approximately 190 g) received a right uninephrectomy or sham operation under light 3% isoflurane anesthesia. Two weeks after the surgery, an osmotic minipump (model 2004; Alzet, Cupertino, CA) was implanted subcutaneously to infuse Aldo. Rats were randomly divided into four groups for 4 weeks: group 1 served as the control group as no pump was implanted in these animals (n=6); group 2, Aldo (0.75 μg/kg body weight per minute)-infused rats (n=6); group 3, Aldo-infused rats with RP (1 mg/kg per day, intraperitoneal [ip]) (n=6); and group 4, Aldo-infused rats with CQ (60 mg/kg per day, ip) (n=6). All groups received 1% NaCl in their drinking water throughout the experimental period.

Systolic blood pressure (SBP) was measured in the conscious state by tail-cuff plethysmography (BP-98A; Softron Co., Tokyo, Japan) at weeks 0 and 4 during the treatment period. Twenty-four-hour urine samples were collected starting after a 24-h acclimatization period in metabolic cages. Urinary protein excretion was determined using enzyme-linked immunosorbent assay (ELISA) kits from Exocell (Philadelphia, PA, USA). Urine and plasma creatinine levels were analyzed using an assay kit (Jiancheng, Nanjing, China).

### Morphological analysis and immunohistochemistry

Kidney sections (3 μm thick) stained with periodic acid-Schiff (PAS) were used to evaluate glomerular lesions, such as glomerular cell proliferation, expansion of the mesangial matrix and segmental glomerulosclerosis. The severity of glomerular injury was determined according to a method described previously [21, 49]. In brief, the severity of injury for each glomerulus was scored from 0 to 4: 0, no lesion; 1, <25% involvement of the glomerulus; 2, 25-50% involvement; 3, 50-75% involvement; and 4, >75% involvement. Fifty glomeruli were analyzed per kidney section. A glomerular sclerosis score (GSS) per animal was calculated by multiplying each severity score (0-4+) with the percentage of glomeruli displaying the same degree of injury and by summing these scores. The above histological analysis was performed in a blind manner to avoid bias. Immunohistochemical stains were performed on formalin-fixed, paraffin-embedded 3-μm sections. Primary antibodies against the following proteins were used: LC3 (1:1000), Nephrin (1:200), and Podocin (1:200). Staining was visualized using horseradish peroxidase-coupled secondary antibodies (Vectastain elite, Vector Labs). All immunohistochemical analyses were repeated at least three times, and representative images are shown.

### Glomerular isolation

Isolation of glomeruli was performed as previously reported [50]. The purity of the glomerular preparation was >95% as determined by light microscopy. Briefly, kidneys were first perfused with a mixture of Dynabeads (diameter 4.5 μm) and iron powder (diameter 6 μm). Tissues were then rinsed on a 70-mm nylon mesh after magnetic treatment to remove small tubular fragments.

### Terminal deoxynucleotidyl transferase dUTP nick-end labeling (TUNEL) staining

Apoptotic cells were detected using the TUNEL assay with an *in situ* apoptosis detection kit (Takara Bio Inc., Tokyo, Japan). Apoptosis was defined as the presence of nuclear condensation via DAPI staining and TUNEL-positive cells within the glomeruli. The percentage of podocytes with TUNEL-positive glomerular cells in formalin-fixed renal tissue was determined by examining at least 30 glomeruli at 400× magnification.

### Transmission electron microscopy

Kidney tissue specimens from rats were fixed in 1.25% glutaraldehyde/0.1 mol/l phosphate buffer and post-fixed in 1% OsO_4_/0.1 mol/l phosphate buffer. Ultra-thin sections of 60 to 80 nm thickness were incised using an Ultracut E ultramicrotome and stained in alcoholic uranyl acetate (10 min) and lead citrate (10 min) before examining the samples on a transmission electron microscope (JEOL JEM-1010, Tokyo, Japan). The slit pore diameter was measured as previously described [51, 52].

### Statistical analyses

Data are expressed as the mean ± standard error of mean (SEM). Comparisons between groups were performed with one-way analysis of variance (ANOVA) followed by Dunnett's multiple comparison tests or Student's t-test. P<0.05 was considered statistically significant.

## Abbreviations

Aldo: aldosterone
RP: rapamycin
CQ: chloroquine;
3-MA: 3-methyladenine
RAAS: renin-angiotensin-aldosterone system
CKD: chronic kidney diseases
mTOR: mammalian target of rapamycin
S6K1: ribosomal protein S6 kinase 1
4EBP-1: eukaryotic initiation factor 4E-binding protein-1
SIRT: NAD+-dependent histone deacetylase
HDAC: histone deacetylase
Tun: tunicamycin
TAUDC: tauroursodeoxycholic acid
ER: endoplasmic reticulum
